# Identification of two immortalized cell lines, ECV304 and bEnd3, for *in vitro* permeability studies of blood-brain barrier

**DOI:** 10.1371/journal.pone.0187017

**Published:** 2017-10-23

**Authors:** Shu Yang, Shenghui Mei, Hong Jin, Bin Zhu, Yue Tian, Jiping Huo, Xu Cui, Anchen Guo, Zhigang Zhao

**Affiliations:** 1 Department of Pharmacy, Beijing Tiantan Hospital, Capital Medical University, Beijing, China; 2 Institute of Disease Prevention and Control of PLA, Beijing, China; 3 Neurology Research, Henry Ford Hospital, Detroit, Michigan, United States of America; 4 Laboratory of Clinical Medicine Research, Beijing Tiantan Hospital, Capital Medical University, Beijing, China; 5 Beijing Key Laboratory of Translational Medicine for Cerebrovascular Disease, Beijing, China; 6 Center of Stroke, Beijing Institute for Brain Disorders, Beijing, China; Nanjing Normal University, CHINA

## Abstract

To identify suitable cell lines for a mimetic system of *in vivo* blood-brain barrier (BBB) for drug permeability assessment, we characterized two immortalized cell lines, ECV304 and bEnd3 in the respect of the tightness, tight junction proteins, P-glycoprotein (P-gp) function and discriminative brain penetration. The ECV304 monoculture achieved higher transendothelial electrical resistance (TEER) and lower permeability to Lucifer yellow than bEnd3. However, co-culture with rat glioma C6 cells impaired the integrity of ECV304 and bEnd3 cell layers perhaps due to the heterogeneity among C6 cells in inducing BBB characteristics. The immunostaining of ZO-1 delivered distinct bands along cell borders on both cell lines while those of occludin and claudin-5 were diffused and weak. P-gp functionality was only proved in bEnd3 by Rhodamine 123 (R123) uptake assay. A permeability test of reference compounds displayed a similar rank order (digoxin < R123 < quinidine, verapamil < propranolol) in ECV304 and bEnd3 cells. In comparison with bEnd3, ECV304 developed tighter barrier for the passage of reference compounds and higher discrimination between transcellular and paracellular transport. However, the monoculture models of ECV304 and bEnd3 fail to achieve the sufficient tightness of *in vitro* BBB permeability models with high TEER and evident immunostaining of tight junction proteins. Further strategies to enhance the paracellular tightness of both cell lines to mimic *in vivo* BBB tight barrier deserve to be conducted.

## Introduction

The blood-brain barrier (BBB), mainly composed of endothelial cells that line brain capillaries, is characterized by the presence of tight junctions and efflux transporter systems. The physical barrier seals the paracellular passage of hydrophilic molecules while efflux transporters like P-glycoprotein (P-gp) restrict the transcellular passage of lipophilic molecules by extruding them back into the blood [1]. BBB limits the entry of the drugs into the brain and the development of *in vitro* predictive models for BBB permeability is important for neurological drug discovery.

Artificial membrane and cell culture models are common methods for BBB permeability studies. Parallel artificial membrane permeability assay (PAMPA), based on porcine brain extract, mimics the lipoidal microenvironment of BBB for passive diffusion transport [2]. Primary culture of endothelial cells isolated from porcine, bovine, rodent and human can closely reproduce the *in vivo* BBB characteristics of tight junction and expression of efflux transporters. However, the primary cell culture is expensive, time-consuming and technique-demanding and thus the immortalized cell lines are developed for BBB permeability studies. Brain capillary endothelial cell lines such as mouse bEnd3, porcine PBMEC/C1-2 and human hCMEC/D3, and non-cerebral cell lines like CaCo-2, MDCK-MDR1 and ECV304 could form the tight paracellular barrier and represent popular cell lines for BBB studies [3–5]. A co-culture with glial cells to simulate the *in vivo* BBB may enhance the barrier function of endothelial cells. In addition, flow-based hollow-fiber models, microfluidic models and human pluripotent stem cells-derived models have been established for BBB studies. However, they require sophisticated expertises and are not in wide usage at present [6].

ECV304, firstly reported as a human umbilical vein endothelial cell line and later proved to exhibit phenotypic characteristics similar to human bladder cancer cell line, is used for BBB studies due to its capacity to generate tight paracellular barrier [7, 8]. It is inducible of BBB characteristics when co-cultured with glial cells like rat glioma C6 cell line [9]. bEnd3, a mouse brain microvascular cell line transformed with Polyoma virus middle T antigen, showed fluorescein permeability and expression of tight junction protein claudin-5 similar to those of primary mouse endothelial cells [10]. It is also characterized by the presence of a variety of transporters including P-gp, glucose transporter (GLUT1) and monocarboxylic acid transporter (MCT1) [11].

To identify cell lines suitable for drug testing of BBB permeability, we evaluated ECV304 and bEnd3 cell lines in the respect of barrier tightness and P-gp function. The ECV304 and bEnd3 monoculture and co-culture models with C6 cells were tested related to tightness through transendothelial electrical resistance (TEER) and permeability for Lucifer yellow. The tight junction proteins occludin, claudin-5 and ZO-1 on both cell lines were detected by immunofluorescence method. P-gp function was assessed using Rhodamine 123 (R123) uptake assay. Additionally, a permeability testing of reference compounds was performed on ECV304 and bEnd3 cell layers.

## Materials and methods

### Materials

ECV304, C6 and bEnd3 cell lines were obtained from American Type Culture Collection (ATCC), Medium 199 (M199), Dulbecco’s modified Eagle’s medium (DMEM), fetal bovine serum (FBS), trypsin (0.25%)-EDTA (0.02%) solution, penicillin-streptomycin solution and Hank’s balanced salt solution (HBSS) were purchased from Hyclone (Logan, UT, USA), Lucifer yellow, quinidine, digoxin and R123 were purchased from Sigma Aldrich (St. Louis, MO, USA), verapamil and propranolol were obtained from Macklin Inc. (Shanghai, China).

### Cell culture

ECV304 and C6 cell lines were incubated with M199 containing 10% FBS and 1% penicillin-streptomycin while bEnd3 cells were grown in DMEM supplemented with 10% FBS and 1% penicillin-streptomycin. Cell culture was performed in a humidified atmosphere of 5% CO_2_ in air at 37°C and the confluent cells were passaged by the trypsin (0.25%)-EDTA (0.02%) solution at a split ratio of 1:5~1:10.

For the generation of mono-culture models, ECV304 or bEnd3 cells were seeded on the upper surface of the membrane in Polyester Transwell inserts (0.4 μM pore size, 6.5 mm diameter, 24 well, Costar, Kennebunk, ME, USA) at a density of 5 × 10^4^ and 8 × 10^4^/cm^2^, respectively. For the generation of co-culture models, C6 cells were seeded on the lower surface of the membrane at a density of 5 × 10^4^/cm^2^ and incubated for 2 h to allow cell attachment before the seeding of ECV304 or bEnd3 cells. The culture medium (0.25 ml in the apical compartment and 1 ml in the basolateral compartment to avoid hydrostatic pressure) was replenished every day.

### TEER measurement

TEER was measured using an Epithelial-volt-ohm-meter (EVOM) with Endohm-12 chamber electrodes (World Precision Instrument, USA). TEER of blank inserts was subtracted from the measured TEER of each model to reflect that of cell layers themselves. Values were expressed as Ω·cm^2^.

### Transport studies

On day 5~7 after seeding cells, the TEER across cell layers was measured before initiating the transport study by replacing the medium in the apical compartment with tested compounds dissolved in HBSS. The permeability of Lucifer yellow (50 μM) was conducted on ECV304 and bEnd3 monoculture or co-culture models with C6 cells while those of other molecules (R123 and digoxin: 5μM, quinidine: 10 μM, propranolol and verapamil: 20 μM) were performed on the monoculture models at 37°C. At indicated time points of 15, 30, 45 and 60 min, the inserts were transferred into new wells containing fresh HBSS to maintain sink conditions. Lucifer yellow (excitation: 430 nM; emission: 540 nM) and R123 (excitation: 485 nM; emission: 535 nM) were measured by a PerkinElmer EnSpire Multimode Plate Reader. Other compounds were analyzed by liquid chromatography tandem mass spectrometer (LC-MS/MS).

The cleared volume was determined by dividing the compound amount in basolateral compartment by the compound concentration in apical compartment. The slope of the cumulative clearance versus time was calculated by linear regression analysis and denoted as permeability coefficient (PE_all_) × surface area of the membrane insert (S, 0.33 cm^2^). The PE_blank_ of blank inserts was included to calculate the PE_cell_ for cell layers themselves using the following equation: 1/PE_cell_S = 1/PE_all_S—1/PE_blank_S.

### Immunofluorescence microscopy

The ECV304 and bEnd3 were seeded on the coverslips (WHB, Shanghai, China) at a density of 3×10^4^/well in 24-well plates. After 3~4 days, the confluent cells were fixed with 4% paraformaldehyde (Solarbio, Beijing, China) for 10~20 min at room temperature. Then the cells were washed in PBS and permeabilized by 0.1% Triton X-100 (Sigma, USA) in 5% normal goat serum (Applygen Technologies Inc., Beijing, China) for 30min. The incubation with a 1:100 dilution of rabbit anti-occludin, ZO-1 (Proteintech, Rosemont, IL, USA) and claudin-5 (ab15106, Abcam, UK) primary antibody was performed at 4°C overnight. After washing in PBS, the cells were treated with secondary antibody (Alexa Fluor 488 conjugated goat anti-rabbit IgG, Molecular Probes, USA) for 30 min at room temperature and then stained with DAPI (Beyotime, Shanghai, China). The coverslips were mounted using fluorescent mounting medium (ZSGB-BIO, Beijing, China) and the images were obtained by a ZEISS LSM 710 confocal microscope (Carl Zeiss AG, Oberkochen, Germany). Negative controls were performed in parallel in the absence of primary antibodies.

### R123 uptake assay

R123 uptake assay was carried out to identify P-gp function. The confluent ECV304 and bEnd3 cells in 24-well plates were exposed to blank HBSS or HBSS containing P-gp inhibitor verapamil (100 μM) for 30 min at 37°C. Then the medium was removed and P-gp substrate R123 (5 μM) dissolved in HBSS alone or with the inhibitor was added to cells and the incubation lasted for 1 h. After replacing in HBSS with or without the inhibitor, the plate was maintained at 37°C for another 2 h. The cells were lysed with 1% Triton X-100 in PBS for 15min at 37°C and the aliquots were removed for R123 detection using a PerkinElmer EnSpire Multimode Plate Reader (excitation: 485 nM; emission: 535 nM). The protein content was determined by a BCA assay kit (Applygen Technologies Inc., Beijing, China). The R123 concentration was expressed as ng/mg protein.

### LC-MS/MS conditions

The analysis was carried out on an Acquity UPLC H-class (Waters, MA, USA) tandem QTRAP 5500 mass system (AB SCIEX, CA, USA). The separation was operated on a Waters Acquity UPLC BEH C_18_ column (2.1 × 50 mm, 1.7 μM particles) under gradient elution with mobile phase A (methanol) and mobile phase B (0.05% formic acid in water) as follows: initial, 10% A; 0 ~ 0.7 min 10% A– 90% A; 0.7 ~ 1.4 min, 90% A; 1.4 ~ 1.5 min, 90% A– 10% A; 1.5 ~ 3min, 10% A. The flow rate was 0.4 ml/min.

The positive electrospray ionization (ESI) was performed with optimal conditions as follows: ion spray voltage, 5500 V; temperature, 550°C; curtain gas, 30 psi; ion source gas 1 and ion source gas 2, 50 psi. The mass spectrometric analysis was operated in multiple reaction monitoring (MRM) mode with ion transitions at *m/z* 325.1 > 172.1 for quinidine, *m/z* 455.2 > 165 for verapamil and *m/z* 260 > 183 for propranolol, respectively. The negative ESI was performed under ion spray voltage of -4500 V for digoxin (*m/z* 779.4 > 649.2).

### Statistical analysis

The values were compared using the analysis of two-tail paired Student’s *t* test. Changes were considered as statistical significance at *p* < 0.05.

## Results

### Tightness measurement in ECV304 and bEnd3 monoculture and co-culture models with C6 cells

**Fig 1:**
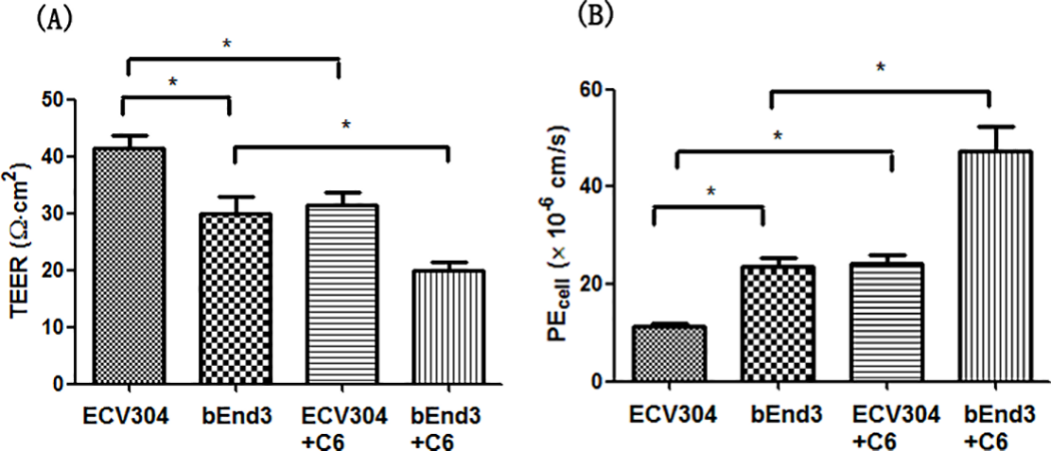
**TEER (A) and permeability to Lucifer yellow (B) in ECV304 and bEnd3 monoculture and co-culture models with C6 cells.** ECV304 demonstrated higher TEER and lower permeability to Lucifer yellow than bEnd3. However, a co-culture of ECV304 or bEnd3 with C6 cells resulted in the decrease of TEER and increase of permeability to Lucifer yellow. Data represent means ± SD (n = 3). * *p* < 0.05.

Transport studies were performed at 5~7 days after the seeding of cells because TEER and permeability to Lucifer yellow reached plateau phase at this period and increasing incubation time will not enhance the tightness of cell layers. As show in Fig 1, the TEER and permeability to Lucifer yellow in ECV304 monoculture model were 41.5 ± 2.12 Ω·cm^2^ and 11.17 ± 0.87 × 10^−6^ cm/s, respectively. The bEnd3 monolayer was looser than ECV304, as reflected by the lower TEER (30 ± 2.83 Ω·cm^2^) and higher permeability to Lucifer yellow (23.68 ± 1.71 × 10^−6^ cm/s). However, when co-cultured with C6 cells, ECV304 showed a 25% reduction of TEER and 2.2-fold increase of permeability to Lucifer yellow, while a 34% reduction of TEER and 2-fold increase of permeability to Lucifer yellow were observed in bEnd3. These data indicated the deleterious effects of C6 cells on the integrity of ECV304 and bEnd3 cell layers.

### Immunostaining of occludin, claudin-5 and ZO-1 in ECV304 and bEnd3 cells

**Fig 2:**
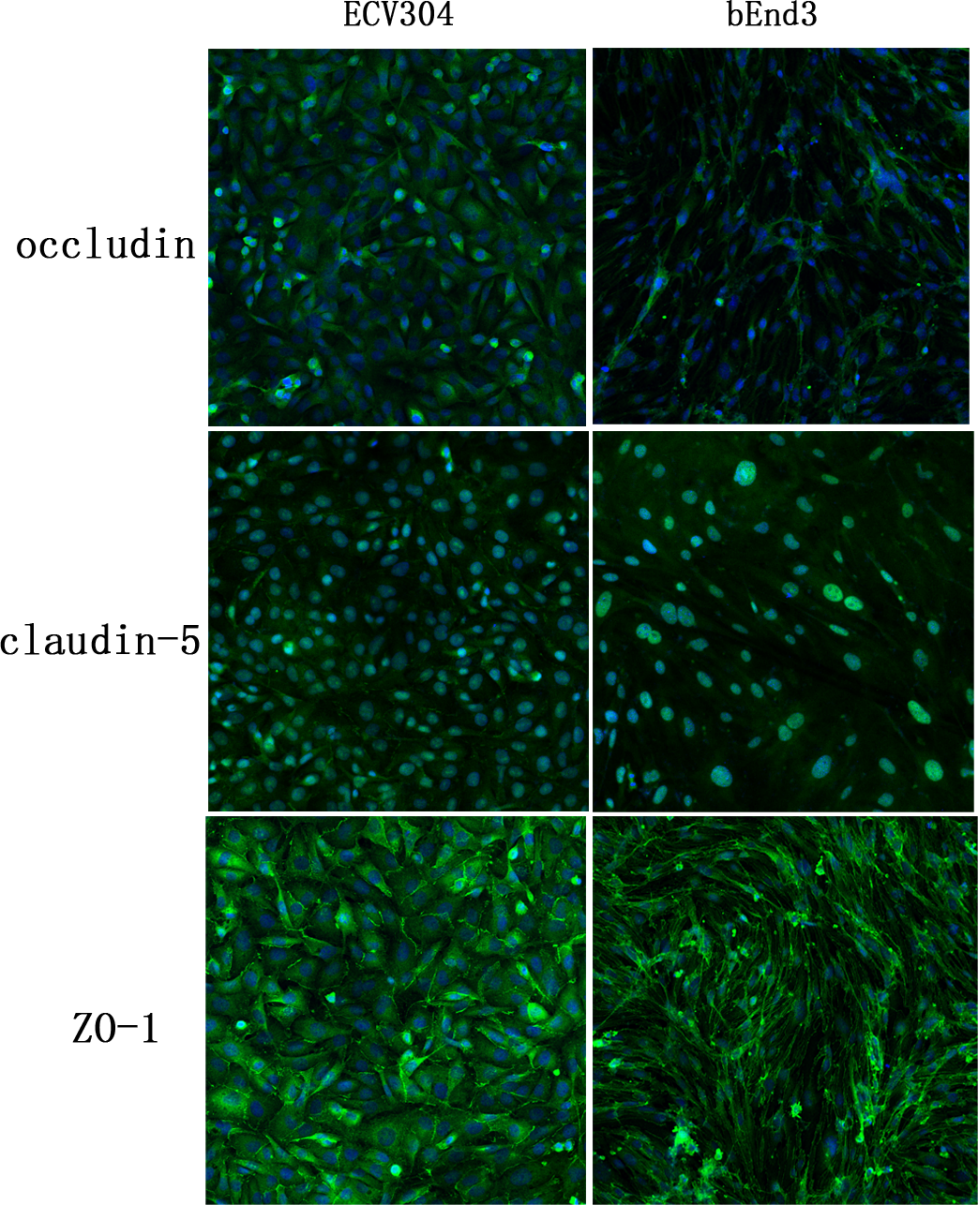
Immunofluorescent staining of tight junction proteins occludin, claudin-5 and ZO-1 in ECV304 and bEnd3 cells. The immunofluorescence of ZO-1 gave distinct strands on cell membrane while the staining of occludin and claudin-5 were diffused and weak in both cell lines. The confocal images were acquired at 20 × magnification.

The tight junction proteins occludin, claudin-5 and ZO-1 are crucial for the restrictive paracellular barrier of BBB. We detected the presence of three tight junction proteins in ECV304 and bEnd3 cells by immunofluorescence method. The staining of ZO-1 was distinct on cell membrane while those of occludin and claudin-5 were diffused and weak (Fig 2).

### P-gp functionality in ECV304 and bEnd3 cells

**Fig 3:**
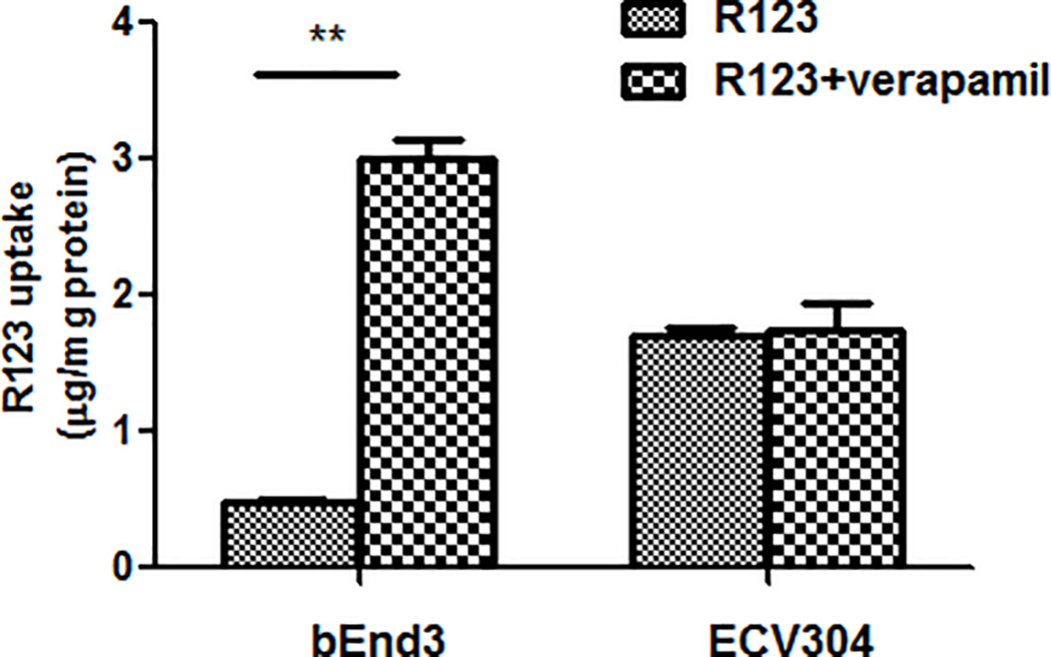
R123 uptake in ECV304 and bEnd3 cells in the absence or presence of P-gp inhibitor verapamil. The P-gp inhibitor verapamil delivered no significant effects on R123 uptake in ECV304 cells but significantly increased R123 uptake in bEnd3 cells in comparison with that in the absence of the inhibitor. Data represent means ± SD (n = 3). ** *p* < 0.01.

Efflux transporter P-gp is an important influential factor on drug permeation across BBB and R123 uptake assay was applied to assess its function in ECV304 and bEnd3 cells. R123 uptake in ECV304 was not significantly changed while that in bEnd3 cells was increased by 6.2-fold by the P-gp inhibitor verapamil versus absence of the inhibitor (Fig 3), which proved the functionality of P-gp in bEnd3 cells.

### Permeability studies in ECV304 and bEnd3 monoculture models

The permeability of reference compounds were validated in ECV304 and bEnd3 monoculture models. In both cell lines, the permeability of the tested compounds achieved a similar rank order: the lowest permeability was observed for digoxin and the highest permeability was obtained for propranolol. A higher permeability of R123 was determined versus that of digoxin. Quinidine and verapamil demonstrated the permeability close to that of propranolol. However, the permeability coefficients of the tested compounds across ECV304 were lower with different degrees than those in bEnd3 probably due to the tighter cell layers of ECV304 versus bEnd3 (Table 1). In addition, the permeability ratio of the transcellular marker propranolol to paracellular marker Lucifer yellow in ECV304 cells was higher than that in bEnd3 cells (2.5 vs 1.2).

**Table 1:** Permeability coefficient values of reference compounds measured in ECV304 and bEnd3 monoculture models.

Compounds	PE_cell_ (× 10^−6^ cm/s)	
	ECV304	bEnd3
**Propranolol**	28.42 ± 1.25	29.68 ± 1.44
**Verapamil**	23.25 ± 0.87	28.37 ± 0.86 *
**Quinidine**	24.46 ± 1.61	26.79 ± 0.68
**R123**	12.38 ± 0.91	22.34 ± 2.8 *
**Digoxin**	3.29 ± 0.16	6.24 ± 0.27 **

Data represent means ± SD (n = 3).

* *p* < 0.05 and

** *p* < 0.01, versus ECV304 cells

## Discussion

To investigate a mimetic BBB model for drug permeability study *in vitro*, we chose two immortalized cell lines, ECV304 and bEnd3 as their permeability are comparable to those of the *in vivo* BBB [8, 12], and assessed their BBB characteristics with respect to tight junction, P-gp function and discriminative brain penetration.

The functional tightness of BBB models are often assessed by two methods: TEER measurement and tracer flux assay [13]. TEER reflects the monolayer barrier to ion movement. Since ions travel across brain endothelium not only through paracellular pathway but also via ion pores or transporters, TEER is not directly translated into the restrictive barrier of the cell layers [14]. More importantly is the permeability to paracelluar markers such as sucrose, sodium fluorescein and Lucifer yellow. In this study, the TEER and permeability of Lucifer yellow measured in ECV304 monolayer were compatible with those published before (41.5 Ω·cm^2^ vs 62 Ω·cm^2^ and 11.17 × 10^−6^ cm/s vs 10.4 × 10^−6^ cm/s) [15]. In comparison with ECV304, the lower TEER and higher permeability of Lucifer yellow in bEnd3 monolayer suggested that the tightness of bEnd3 cell layers was weaker than that of ECV304.

It is known that the astrocytes or glioma cells induce and strengthen BBB functions of brain endothelial cells via a range of released factors, including transforming growth factor-β (TGFβ), glial-derived neurotrophic factor (GDNF) and basic fibroblast growth factor (bFGF) [16–18]. However we here showed that a co-culture with rat C6 glioma cells impaired the integrity of ECV304 and bEnd3 cell layers. This may be attributed to the heterogeneity among C6 cells in inducing BBB characteristics. It has been shown that C6 cells also secreted the cytokines like tumor necrosis factor-alpha (TNF-α) to open the BBB barrier [19]. The destructive factors involved in this study deserved to be further elucidated.

Tight junction is formed by a complex of tight junction proteins including transmembrane proteins occludin, claudin-5 and cytoplasmic protein ZO-1. Occludin and claudin-5 interact with membrane proteins on adjacent cells and form the backbone of tight junctions while ZO-1 links transmembrane proteins to actin cytoskeleton for the maintenance and regulation of paracellular barrier. Loss of these important tight junction proteins may result in increased barrier permeability or breakdown of BBB [20]. Our study revealed the presence of the occludin, claudin-5 and ZO-1 in ECV304 and bEnd3 cells by immunofluorescence method. The immunofluorescence of ZO-1 gave distinct strands on cell membrane while the staining of occludin and claudin-5 were diffused and weak. These data suggested that the paracellular barrier of cell lines is not as tight as that of primary rat brain endothelial cells which exhibited distinct and continuous staining bands of tight junction proteins along cell borders [21]. Strategies of co-culture with glial cells or the addition of enhancers like hydrocortisone or CPT-cAMP deserved to be conducted for improving the tightness of cell lines.

P-gp is an extensively characterized efflux protein that influences the rate and extent of drug penetration into the brain by pumping compounds back into the blood. Molecular detection at mRNA or protein levels and functionality assays through cellular uptake experiment or bi-directional transport studies are explored to identify transport proteins [22]. In this study, R123 uptake assay was used to assess the function of P-gp in ECV304 and bEnd3 cells. The results showed that the P-gp inhibitor verapamil significantly increased R123 uptake in bEnd3 cells but having no such effects in ECV304 cells, indicating the active P-gp function in bEnd3 cells. Uptake assay in endothelial cells like bEnd3 also represents a useful means for evaluating the capacity of tested compounds to penetrate into the brain [23, 24].

Discriminative brain penetration is an important feature of BBB and compounds with different permeability were evaluated in ECV304 and bEnd3 cells. In both cell lines, the lowest permeability value was observed for P-gp substrate digoxin and the highest permeability was obtained for lipophilic marker propranolol, which is consistent with previous studies in a triple-culture model of primary rat brain endothelial cells, astrocytes and pericytes [21]. Since ECV304 demonstrated no detectable P-gp activity, the barrier for digoxin transport may be attributed to its intrinsic low membrane permeability. This may also account for low permeability of P-gp substrate R123 [25]. Verapamil and quinidine are high permeable P-gp substrates, and for verapamil at high concentration of 10 μM, passive diffusion may overwhelm P-gp efflux [14, 26, 27]. Both compounds delivered relatively high permeability across ECV304 and bEnd3 cell layers. However, ECV304 developed tighter barrier for the flux of tested compounds than bEnd3. In addition, the permeability ratio of propranolol to hydrophilic marker Lucifer yellow in ECV304 was higher than that in bEnd3 (2.5 vs 1.2), which is in agreement with previous reports that bEnd3 was not able to effectively discriminate between paracellular and transcellular transport [11, 28].

## Conclusions

The ECV304 developed tighter monolayers and better discriminated between transcellular and paracellular transport than bEnd3 while P-gp function was only identified in bEnd3. However, the monolayers of both cell lines lack the sufficient tightness of *in vitro* BBB permeability models with the TEER over 1000 Ω·cm^2^ and evident immunostaining of tight junction proteins [29]. Further strategies to enhance their paracellular tightness to mimic *in vivo* BBB tight barrier need to be carried out.
